# Chronic Pulmonary Aspergillosis Following Nontuberculous Mycobacterial Infections: An Emerging Disease

**DOI:** 10.3390/jof6040346

**Published:** 2020-12-08

**Authors:** Pakpoom Phoompoung, Methee Chayakulkeeree

**Affiliations:** Division of Infectious Diseases and Tropical Medicine, Department of Medicine, Faculty of Medicine Siriraj Hospital, Mahidol University, 2 Wanglang Road, Bangkoknoi, Bangkok 10700, Thailand; pakpoom.pho@mahidol.ac.th

**Keywords:** aspergillosis, non-tuberculous mycobacteria, *Aspergillus*, pulmonary infection, fungal infection

## Abstract

Chronic pulmonary aspergillosis (CPA) following nontuberculous mycobacterial (NTM) lung disease is being increasingly recognized, especially in countries where tuberculosis is not endemic, with an incidence rate of 3.9–16.7%. NTM lung disease has been identified as a predictor of mortality in CPA patients. The major risk factors for NTM-associated CPA include fibrocavitary NTM lung disease, the presence of pulmonary emphysema, and high-dose corticosteroid use. The onset of CPA is 1.5–7 years following the diagnosis of NTM lung disease. The diagnosis can be made using standard criteria; however, serological diagnosis using *Aspergillus* precipitin has demonstrated a higher sensitivity and specificity when compared with fungal culture from respiratory specimens. Treatment is challenging since rifampicin and oral triazoles should not be used concomitantly. The prognosis is poor, and the factors associated with worse prognosis are corticosteroid use and high C-reactive protein level.

## 1. Introduction

Chronic pulmonary aspergillosis (CPA) is a slowly progressive pulmonary infection that is caused by *Aspergillus* species, and which results in destruction of lung parenchyma [1]. This disease entity is a semi-invasive form of pulmonary aspergillosis that is classified into the following three forms: chronic cavitary pulmonary aspergillosis, chronic fibrosing pulmonary aspergillosis, and subacute invasive pulmonary aspergillosis (formerly chronic necrotizing pulmonary aspergillosis) [2]. CPA can be complicated in patients with various immunocompromising conditions, and in those with structural lung diseases [3]. It is also one of the most common complications following treatment of pulmonary tuberculosis (TB) [4,5]. The prevalence rate of CPA following TB was reported to range from 6–22% [6,7]. Among all patients diagnosed with CPA, TB was identified as the most common pre-existing lung condition (17–93%) [8,9]. In contrast, an association between pulmonary nontuberculous mycobacterial (NTM) infections and CPA has not been well demonstrated. Despite the fact that a number of NTMs can cause chronic pulmonary diseases that result in lung destruction [10], cases that developed CPA after NTM were traditionally only rarely reported. The diagnosis of this condition was often delayed, and management was a challenge due to drug–drug interaction, which resulted in worse prognosis. In this review, we highlight the importance of NTM as one of the emerging risk factors for CPA. The epidemiology, pathogenesis, risk factors, clinical manifestations, management, and prognosis are also discussed in detail.

## 2. Epidemiology

The incidence of CPA following NTM lung diseases varied from study to study, with a reported incidence of 3.9–16.7% [11,12]. However, this range of incidence may be an underestimate, due to the uncertain diagnosis of NTM lung diseases. In TB-endemic countries, up to 93% of patients with CPA were diagnosed in association with pre-existing pulmonary tuberculosis [8,13,14]. However, in countries with a lower incidence of TB, such as the United Kingdom, pulmonary NTM was identified as the second leading cause of CPA (14.9%) following pulmonary tuberculosis (15.3%) [9].

An association between NTM and *Aspergillus* is being increasingly recognized, as evidenced by the growing number of case series and multiple case reports [15,16,17,18]. Since 1985, multiple case series of CPA following pulmonary NTM infection have been described, and this has increased interest in the possible association between these two common diseases. To illustrate this, Bollert and colleagues reported three patients who suffered from chronic obstructive pulmonary disease (COPD) and *Mycobacterium malmoense* lung infection [16]. They all had superimposed *Aspergillus* infection after NTM lung disease, which led to CPA, lung destruction, and death. Maliwan and colleagues reported 263 patients who had been diagnosed with *Mycobacterium kansasii* pulmonary infection [15]. They found seven patients (2.7%) who developed CPA with an onset of 2–10 years after NTM infection. The mortality rate among those seven patients was 71%. Johnston and colleagues reported 11 patients with *Mycobacterium xenopi* lung infection, and eight of them were subsequently diagnosed with pulmonary aspergilloma at 1–4 years after diagnosis of NTM lung disease [17].

Kunst and colleagues reported the first study to confirm NTM infection as one of the significant risk factors for CPA. They performed a case-control study that compared 31 patients with underlying bronchiectasis who had NTM infections with 61 non-NTM patients as a control group [19]. The most common NTM species isolated in that study was *Mycobacterium avium* complex (MAC), followed by *Mycobacterium kansasii*. The rate of *Aspergillus* serology positivity was higher in those with NTM infections (33.3%) than in controls (9.8%). In addition, six patients with NTM infection demonstrated radiological features suggestive of *Aspergillus*-related lung diseases, whereas those features were not presented in any of the control group patients. Their multivariate analysis revealed NTM to be an independent risk factor for CPA in bronchiectasis patients (odds ratio (OR): 5.1, 95% confidence interval (CI): 1.5–17).

## 3. Pathogenesis

The causative relationship between NTM and *Aspergillus* lung infection is not sufficiently well understood. However, many hypotheses have been proposed to explain the association between these two pathogens. NTM-infected patients usually receive long-term broad-spectrum antibiotics that increase the risk of fungal colonization due to selective pressure. This may eventually lead to *Aspergillus* lung infection, especially in those with underlying structural lung diseases. Furthermore, NTM lung disease is commonly diagnosed in immunocompromised patients, especially those with chronic lung diseases, and in patients who concomitantly received immunosuppressive agents, which increases susceptibility to *Aspergillus* infections [20]. A percentage of patients may develop NTM infection following treatment of tuberculosis [21,22], and tuberculosis itself increases the risk of *Aspergillus* infection. Moreover, NTM lung disease results in destructive lung lesions, including lung cavitation, that leads to an increasing incidence of CPA [21,22].

## 4. Risk Factors

Risk factors for CPA following NTM diseases were reported by some single-center retrospective studies. A retrospective study from Japan that included 82 patients with NTM pulmonary diseases reported nine patients (11%) with CPA complicating NTM infection [23], while cavitary NTM infection (OR: 3.49, 95% CI: 1.48–9.24) and steroid use (OR: 2.75, 95% CI: 1.05–7.20) were the independent risk factors for the development of CPA. The limitation of that study was its relatively small sample size. In addition, four out of nine patients had coexisting CPA and NTM lung infection. As a result, one could argue that these two factors could be predictors of NTM-*Aspergillus* coinfection, rather than predictors of CPA following NTM lung diseases.

However, these predictors were confirmed by Jhun and colleagues in 2017 [24]. They performed a larger retrospective study in South Korea that investigated risk factors for CPA in patients with NTM lung diseases, and included 566 patients with NTM lung infection. The most common NTM species in that study was MAC (65.9%), followed by *M. abscessus* complex (20.1%). Among all cases, 41 patients (7.2%) developed CPA following NTM infection, with a median onset of 18 months. Multivariate analysis showed systemic corticosteroid use to be the strongest predictor of CPA (OR: 15, 95% CI: 4.74–47.43), followed by fibrocavitary NTM disease (OR: 7.93, 95% CI: 3.24–19.41). Other predictors identified in that study included old age, male gender, low body mass index (BMI < 18.5 kg/m^2^), pulmonary emphysema, and *M. abscessus* complex infection.

Furuuchi and colleagues retrospectively investigated risk factors for CPA in patients with MAC lung disease [24]. That study found multiple independent risk factors, including pulmonary emphysema, baseline steroid use, serum albumin level <3.5 g/dL, and the presence of cavitary lesions. Patients having more than one risk factor were more likely to develop pulmonary aspergillosis, with a 5-year incidence rate of 31%, compared to 2.2% incidence in patients who had zero to one risk factors.

Shirai and colleagues studied the clinical significance of *Aspergillus* precipitating antibody in patients with pulmonary MAC disease. Subsequent CPA was commonly observed in patients who had positive antibody at the time of NTM diagnosis (60% vs. 10.8%, *p* < 0.001). This finding may suggest previous fungal colonization as one of the important risk factors for CPA following NTM lung disease. Of note, positive antibody patients had significantly higher frequencies of pulmonary emphysema (60% vs. 13.5%, *p* < 0.001) [25].

In summary, the strongest predictors of CPA following pulmonary NTM disease are fibrocavitary NTM lesions, high-dose steroid use, and the presence of pulmonary emphysema [23,24,26]. Patients with fibrocavitary NTM lung disease are more likely to develop CPA than patients with nodular bronchiectasis. This finding was also observed in patients with tuberculosis, whereas CPA was significantly more common in patients who had residual TB cavitation [5]. This can easily be explained by the fact that *Aspergillus* colonization is more prevalent in patients who have pre-existing chronic lung cavities. Prednisolone equivalent dosage of 10 mg/day, or a cumulative dose of more than 700 mg of oral prednisolone, was the corticosteroid dosage significantly associated with CPA from the aforementioned studies [23]. Long-term inhaled corticosteroid use is also a potential risk factor. Corticosteroids impair multiple host immune systems, including innate (phagocyte) and adaptive (lymphocyte) immunity [27], which leads to an inability to kill fungi. Corticosteroids also increase *Aspergillus* colonization and the growth of *Aspergillus* hyphae [27,28,29].

## 5. NTM Species and Risk of CPA

The association between different NTM species and incidence of CPA has not been fully elucidated. Most studies have reported MAC as the most common NTM species that causes CPA [24,30,31]. Nevertheless, no study has conclusively demonstrated MAC as the main predictor of *Aspergillus* infection, compared with other NTM species. The reported predominance of MAC infection preceding CPA may be explained by two reasons. First, most studies were performed in Japan where MAC is the most common causative agent in NTM lung diseases [32]. Second, MAC is the most common NTM species that causes fibrocavitary NTM lung disease. Since we know that fibrocavitary lesion is strongly associated with CPA, it could indirectly result in an increased incidence of CPA following MAC lung disease.

In contrast, *M*. *abscessus* was the only NTM species found to be strongly associated with CPA. Jhun et al. reported *M. abscessus* complex to be an independent predictor of CPA [26], whereas MAC did not significantly increase the risk. Infections with *M. abscessus* subsp. *abscessus* (OR: 5.12, 95% CI: 1.66–15.78) or *M. abscessus* subsp*. masilliense* (OR: 5.53, 95% CI: 1.94–15.78), the two species within the *M. abscessus* complex, increased the risk of CPA. Of note, the incidence of CPA was much higher in patients with *M. abscessus* complex lung infection than in those with pulmonary MAC disease (14.9% vs. 5.1%). The authors observed that the patients who suffered from *M. abscessus* complex lung infection usually did not respond well to anti-mycobacterial therapy, and required a longer course of antimicrobial treatment. Therefore, patients who had *M. abscessus* infection may have undergone CPA testing more frequently than those who had MAC lung disease. The association between *M. abscessus* and *Aspergillus* lung infection is important because *M. abscessus* is becoming more prevalent in many countries, and is the most common etiology of pulmonary NTM diseases in Southeast Asia, and the second most common etiology in East Asian people [33,34,35,36]. Therefore, further studies are urgently needed to investigate whether *M. abscessus* infection increases the risk of *Aspergillus*-related lung diseases, and to improve our understanding of how to manage this syndrome.

## 6. Clinical Manifestation and Diagnosis

The onset of CPA disease is 1.5–7 years after NTM lung infection [12,23,24,30], which is quite similar to the natural history of CPA following TB disease [7]. A diagnosis of CPA following pulmonary NTM infection can be made using the general CPA diagnostic guidelines, which require a combination of clinical characteristics, a consistent appearance in thoracic tomography, direct evidence of Aspergillus infection or immunological response to Aspergillus spp., and exclusion of alternative diagnoses. The common symptoms reported in the literature were fever and weight loss [30]. Hemoptysis was observed in few patients. Since the clinical manifestations of the two diseases are indistinguishable, the microbiological and radiological findings play an important role in making the final diagnosis.

**Figure 1:**
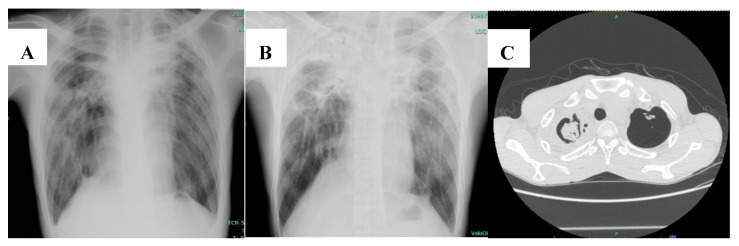
Chest imaging of a 37-year-old man diagnosed with chronic pulmonary aspergillosis following pulmonary *Mycobacterium avium* infection. (**A**) Chest radiograph showed cavitary lesion at right upper lobe (pulmonary *Mycobacterium avium* infection). (**B**) Chest radiograph 1 year later showed thickening of pre-existing cavities, with pericavitary infiltration (chronic pulmonary aspergillosis). (**C**) Computed tomography of the chest showed a fungal ball in the pre-existing cavity.

Common radiological findings that increase the possibility of CPA in NTM-infected patients include thickening of pre-existing lung cavities, the presence of a fungal ball, and infiltration surrounding the cavities [30]. To better illustrate this, Figure 1 shows the chest imaging of a patient who was diagnosed with CPA following pulmonary NTM disease caused by *M. avium*.

Concerning the microbiological diagnosis, the sensitivity of serology (*Aspergillus* precipitin) seems to be better than fungal culture from respiratory specimens (80% vs. 50% in one study) [24]. Moreover, culture positivity of *Aspergillus* species in patients with pulmonary NTM does not always indicate CPA. There was a high rate of *Aspergillus* positive cultures concomitant with positive mycobacterial cultures (35.7% in one study) [37]. *Aspergillus* was more commonly concomitantly detected with NTM infection than with TB (42.9% vs. 25%).

Furuuchi and colleagues performed a retrospective study in 329 patients with pulmonary MAC, with a median follow-up of 3.7 years [31]. Forty patients (12.2%) were culture positive for *Aspergillus* spp.; however, only nine of those 40 patients (22.5%) were diagnosed with CPA. *Aspergillus fumigatus* was the most common etiologic agent of CPA (5/8, 62.5%) when compared with other *Aspergillus* species (4/32, 12.5%). Of note, *Aspergillus niger* was often identified as a common colonizer in NTM-infected patients. This may be explained by the fact that *A. fumigatus* has smaller fungal conidia, which can more easily penetrate to the alveoli and cause lung disease [38]. Therefore, diagnosis of CPA in NTM-infected patients who had positive fungal cultures for non-*fumigatus Aspergillus* spp., without other supporting evidence, should be made with caution.

## 7. Treatment

Treatment of CPA following NTM lung disease remains challenging. Treatment is similar to that of treatment for CPA in general, and the antifungals of choice are oral mold-active triazoles (itraconazole or voriconazole) for a minimum duration of 4–6 months [3]. Treatment of CPA following NTM lung diseases was more challenging compared with CPA following pulmonary TB, since most patients were still on antimycobacterial therapy. This led to drug–drug interaction between oral triazoles and antimycobacterial agents. Most patients had pulmonary MAC infection, which required rifamycin (rifampicin or rifabutin) treatment. Rifamycin is a strong CYP3A4 inducer, so co-administration of rifamycin and itraconazole, or voriconazole, would lead to a significantly lower level of the antifungal, which would result in treatment failure [39]. Although a novel triazole (isavuconazole) has been approved for treatment of invasive aspergillosis, data in CPA is still limited, and the challenge of drug–drug interaction between isavuconazole and rifamycin still exists. Posaconazole and amphotericin B can be used as a third-line treatment for CPA, with less interaction with rifamycin [40]. Although rifampicin may reduce the blood level of posaconazole, it may be used with caution if there is no other antifungal agent available. Amphotericin B is not practical for long-term use in this setting, due to significant nephrotoxicity. Echinocandins (micafungin and caspofungin) can be used as alternative agents due to less significant drug–drug interaction and less toxicity [41,42]. However, their use may be limited by the availability of only intravenous preparation. There are novel antifungal agents under development that have activity against *Aspergillus* spp., with less CYP450 drug interaction, that may be able to be used together with rifamycin for treatment of CPA; however, these are future antifungals and more data is required [43].

Therapeutic drug monitoring (TDM) may have a role in patients who are prescribed rifampicin co-administered with a triazole. Using TDM, Moon and colleagues studied the effect of rifamycin (rifampicin or rifabutin) and itraconazole co-administration in 66 patients who had CPA following NTM lung disease [44]. The itraconazole serum concentration was significantly lower in patients receiving rifampicin or rifabutin. However, there were no significant differences in serum itraconazole concentrations between the patients treated with itraconazole and rifampicin, and those treated with itraconazole and rifabutin. They concluded that concomitant use of rifampicin or rifabutin and itraconazole should be avoided in patients with CPA and coexisting NTM lung disease.

**Figure 2:**
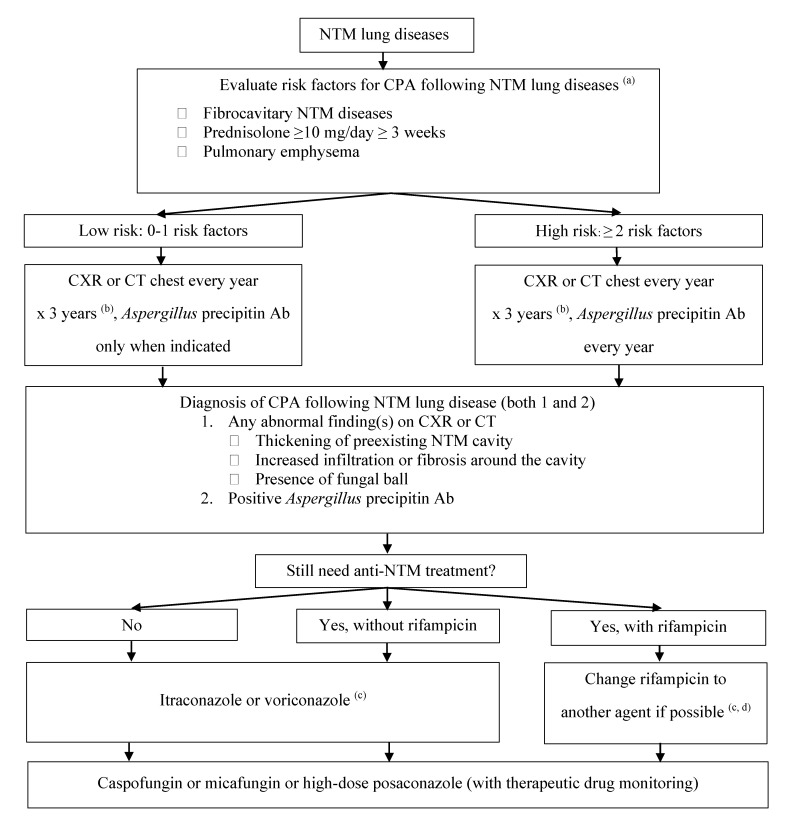
Proposed algorithm for diagnosis and treatment of CPA following NTM lung diseases. ^(^^a^^)^ The strongest risk factors were selected based on previous studies. ^(^^b^^)^ Three years were recommended based on previous data that most cases of CPA occurred within 3 years after NTM diagnosis. ^(^^c^^)^ Triazoles should be started 2 weeks after rifampicin discontinuation. ^(^^d^^)^ Rifampicin should be discontinued to avoid drug interaction if possible, since prognosis relies on CPA rather than NTM lung diseases. **Abbreviations:** NTM, non-tuberculous mycobacteria; CPA, chronic pulmonary aspergillosis; CXR, chest X-ray; CT, computed tomography.

Since novel regimens without rifampicin have been used successfully for NTM lung diseases caused by different NTM species, we suggest that rifampicin should be avoided in this situation. In the case of pulmonary NTM diseases in which rifampicin is one of the drugs in the standard regimen (e.g., MAC, *M. kansasii*), alternative agents of anti-mycobacterial regimen should be selected, based on the recommended guideline for pulmonary NTM disease [45,46]. For example, in patients with pulmonary MAC or *M*. *kansasii* disease, rifampicin could be substituted with fluoroquinolones (e.g., moxifloxacin). However, in cases where rifampicin cannot be discontinued, intravenous echinocandins should be considered. High-dose oral posaconazole with TDM may also be considered for long-term therapy. Figure 2 demonstrates a proposed practical algorithm for the diagnosis and management of CPA following NTM disease.

## 8. Prognosis

The outcome of NTM-associated CPA is usually poor [16,47]. NTM-infected patients with CPA had a poorer prognosis when compared with NTM-infected patients without evidence of aspergillosis. CPA has been demonstrated as a predictor of mortality in patients with NTM lung disease. Zoumot and colleagues studied MAC infection in non-cystic fibrosis bronchiectatic patients [11], and compared the clinical characteristics between survivors and non-survivors. CPA was significantly more common in non-survivor patients compared to survivors (87.5% vs. 12.5%, respectively; *p* < 0.001). Fukushima and colleagues retrospectively studied long-term outcomes of MAC lung disease, and reported CPA to be an independent risk factor for mortality (OR: 8.552, 95% CI: 1.335–54.77) [48]. Takeda and colleagues compared the one-year mortality rate between NTM-infected patients with and without CPA [23], and mortality was found to be significantly higher in patients who had CPA as a complication (22.2% vs. 1.4%, respectively; *p* = 0.031). Of note, the most common cause of death was chronic respiratory failure. The same study also compared mortality between patients who had CPA from any cause and patients who had CPA following NTM lung disease. The mortality rate did not differ between groups (15.6% all CPA vs. 22.2% CPA following NTM, respectively). This finding differed from that from a study by Lowes and colleagues, which identified pre-existing NTM lung disease as an important predictor of high mortality in patients with CPA (hazard ratio [HR]: 2.07, 95% CI: 1.22–3.52) [49].

A retrospective study performed in Japan reported systemic corticosteroid use (HR: 3.32, 95% CI: 1.23–9.51) and C-reactive protein levels more than 5 mg/dL (HR: 8.96, 95% CI: 2.15–62.9) as the factors independently associated with high overall mortality in patients who had CPA following NTM lung infection [50]. In addition to corticosteroid use increasing the risk of CPA following NTM lung disease, it also increased the overall mortality rate. Therefore, corticosteroid use in NTM patients should be avoided or tapered to the lowest possible dose.

When comparing the prognosis of CPA following pulmonary TB, with the prognosis of CPA following NTM diseases, the prognosis was poorer in NTM patients. The two-year survival rates were reported to be 83% and 62% in CPA patients who had pre-existing TB and NTM, respectively [49]. The higher mortality rate may be explained by the immunocompromised status of, and the immunosuppressive agent used in, the CPA following NTM group. In addition, drug–drug interaction with rifampicin was commonly noticed in NTM patients, since most patients were still on anti-mycobacterial agents. Table 1 compares various factors between CPA following pulmonary TB, and CPA following pulmonary NTM disease.

## 9. Conclusions

CPA following NTM lung disease is an emerging infectious pulmonary disease that is associated with a higher mortality rate. Patients with fibrocavitary disease are at highest risk. Corticosteroid use is the strong predictor of CPA in NTM-infected patients, and is also associated with an unfavorable prognosis. Early diagnosis via *Aspergillus* precipitin antibody screening is recommended in high-risk-group patients. Rifampicin should be avoided, if possible, since the prognosis relied on CPA rather than NTM disease.

## 10. Future Research

This review summarizes various aspects of CPA following NTM disease based on all existing data reported in the literature. However, most studies were single-center and retrospective in nature. Previous studies focused only on epidemiology, risk factors, and prognosis. Since diagnosis of CPA following NTM disease is usually delayed, and effective treatment has not been well defined, further prospective or controlled studies focused on diagnostic methods and treatment strategies are needed to help clinicians improve the management of this complex disease.

## Figures and Tables

**Table 1:** Comparison between CPA following pulmonary TB, and CPA following NTM lung disease [5,6,7,11,12,23,24,26,37,49].

Parameter	CPA Following Pulmonary TB	CPA Following NTM Lung Disease
Incidence	Higher	Lower
Host	Mostly immunocompetent	Immunocompetent and immunocompromised
Risk factors	Residual cavitation	Fibrocavitary disease Prednisolone ≥ 10 mg/day ≥ 3 weeks Pulmonary emphysema
Microbiological diagnosis	*Aspergillus* precipitin or fungal culture	*Aspergillus* precipitin is preferred Fungal culture had lower specificity due to *Aspergillus* colonization
Treatment	Drug interaction is less concerning since most CPA cases occurred after anti-mycobacterial agent discontinuation	Drug interaction is of more concern since most CPA cases occurred while receiving anti-mycobacterial agents
Prognosis	Better	Poorer

**Abbreviations:** CPA, chronic pulmonary aspergillosis; TB, tuberculosis; NTM, non-tuberculous mycobacteria.
